# IL-22 signaling promotes sorafenib resistance in hepatocellular carcinoma via STAT3/CD155 signaling axis

**DOI:** 10.3389/fimmu.2024.1373321

**Published:** 2024-03-25

**Authors:** Junzhang Chen, Shiran Sun, Hui Li, Xiong Cai, Chidan Wan

**Affiliations:** ^1^ Department of Hepatobiliary Surgery, Union Hospital, Tongji Medical College, Huazhong University of Science and Technology, Wuhan, China; ^2^ Department of Hepatobiliary Pancreatic Tumor Center, Chongqing Key Laboratory of Translational Research for Cancer Metastasis and Individualized Treatment, Chongqing University Cancer Hospital, Chongqing, China

**Keywords:** hepatocellular carcinoma, sorafenib resistance, IL-22, CD155, NK cell

## Abstract

**Introduction:**

Sorafenib is currently the first-line treatment for patients with advanced hepatocellular carcinoma (HCC). Nevertheless, sorafenib resistance remains a huge challenge in the clinic. Therefore, it is urgent to elucidate the mechanisms underlying sorafenib resistance for developing novel treatment strategies for advanced HCC. In this study, we aimed to investigate the role and mechanisms of interleukin-22 (IL-22) in sorafenib resistance in HCC.

**Methods:**

The *in vitro* experiments using HCC cell lines and *in vivo* studies with a nude mouse model were used. Calcium staining, chromatin immunoprecipitation, lactate dehydrogenase release and luciferase reporter assays were employed to explore the expression and roles of IL-22, STAT3 and CD155 in sorafenib resistance.

**Results:**

Our clinical results demonstrated a significant correlation between elevated IL-22 expression and poor prognosis in HCC. Analysis of transcriptomic data from the phase-3 STORM-trial (BIOSTORM) suggested that STAT3 signaling activation and natural killer (NK) cell infiltration may associate sorafenib responses. STAT3 signaling could be activated by IL-22 administration in HCC cells, and then enhanced sorafenib resistance in HCC cells by promoting cell proliferation and reducing apoptosis *in vitro* and *in vivo*. Further, we found IL-22/STAT3 axis can transcriptionally upregulate CD155 expression in HCC cells, which could significantly reduce NK cell-mediated HCC cell lysis in a co-culture system.

**Conclusions:**

Collectively, IL-22 could contribute to sorafenib resistance in HCC by activating STAT3/CD155 signaling axis to decrease the sensitivities of tumor cells to sorafenib-mediated direct cytotoxicity and NK cell-mediated lysis. These findings deepen the understanding of how sorafenib resistance develops in HCC in terms of IL-22/STAT3 signaling pathway, and provide potential targets to overcome sorafenib resistance in patients with advanced HCC.

## Introduction

Hepatocellular carcinoma (HCC) represents a major global health challenge, being one of the most common and lethal forms of cancer (1). Numerous data have shown that over half of patients are initially diagnosed with HCC at an advanced stage (2). Sorafenib is the first FDA-approved tyrosine kinase inhibitor (TKI) for use in advanced HCC and is recommended as the first-line modality for treating patients with advanced HCC. Sorafenib can interfere the transduction of extracellular-signal-regulated kinase (ERK) signaling and inhibit cancer cell proliferation through downregulating the expression of the isoforms of serine/threonine kinases Raf, Raf-1, and B-Raf (3). Moreover, sorafenib is able to enhance natural killer (NK) cell-mediated antitumor immunity as well, which closely correlates with its therapeutic efficacy in HCC (4). Although about 30% of patients with advanced HCC may benefit this sorafenib treatment, it is unavoidable to develop drug resistance in cases, severely impacting survival outcomes (5). Therefore, it is imperative and urgent to further investigate the mechanisms responsible for sorafenib resistance in HCC, which will help to develop innovative therapeutic modalities to conquer sorafenib resistance in patients with advanced HCC.

Signal transducer and activator of transcription 3 (STAT3), which is a transcription factor transduce signaling upon environmental cytokines and growth factors stimuli, has been well demonstrated playing crucial roles in hepatocarcinogenesis and emerging as a potential therapeutic target for HCC. Aberrant STAT3 signaling exerts oncogenic effects by promoting tumor proliferation, survival, chemoresistance, invasion, metastasis, angiogenesis, immunosuppression, and tumor-related inflammation (6). Among the many cytokines mediate STAT3 phosphorylation, interleukin-22 (IL-22)/STAT3 signaling recently has gained a lot of concern owing to dichotomous effect in HCC observed. Briefly, an upregulation of IL-22 in the serum or tumor tissue of HCC patients is common. What’s more, IL-22 levels have been correlated with HCC progression and a worse prognosis (7–9). While, high serum IL-22 level predicts better survival after TACE treatment (10). We assume that this dichotomous effect in clinical is relevant in various treatment options.

Data from extensive studies have suggested that STAT3 signaling promotes sorafenib resistance in HCC by linking cytokines and growth factors stimuli with target genes transcription regulation (11, 12). Interestingly, a most recent study uncovered that T cell-derived IL-22 can confer breast cancer cell resistance to NK cell-meditated lysis and thereby induce immune escape, causing lung metastasis (13). Although this previous study did not determine whether IL-22 activated STAT3 in breast cancer cells to reduce their sensitivity to NK cell-meditated cytotoxicity, solid evidences confirm that tumor cell-intrinsic STAT3 activation can impair NK cell antitumor immunity via intricate mechanisms (14). Therefore, we showed much interest in the relationship between tumor cell-intrinsic IL-22/STAT3 axis and NK cell-meditated killing in sorafenib resistance in HCC.

In this study, we investigated the effects of IL-22 on the sorafenib-mediated direct cellular toxicity to HCC cells and NK cell-mediated lysis of HCC cells *in vitro* and *in vivo*. The current study may expand our understanding of the mechanisms for sorafenib resistance in HCC, and helps to develop more effective strategies to treat HCC.

## Materials and methods

### Clinical tissue collection

HCC tissue samples and adjacent non-tumor liver tissues were collected from patients who underwent surgical resection at Chongqing University Cancer Hospital. All tissue samples were immediately frozen in liquid nitrogen post-resection and stored at -80°C until further use. For immunohistochemical analysis, a portion of each sample was fixed in formalin and embedded in paraffin. Prior to surgery, informed consent was obtained from all patients involved in the study. The consent process included comprehensive information about the study’s purpose, the nature of the tissue collection, and the potential use of the samples for research. Patients were assured of their right to confidentiality and the option to withdraw from the study at any point without affecting their medical care. This study was conducted in accordance with the Declaration of Helsinki and approved by the Institutional Review Board (IRB) and Ethics Committee of Chongqing University Cancer Hospital. The committee reviewed and approved the study protocol, consent forms, and all procedures involving human and animal subjects.

### Cell lines and culture

Human hepatocellular carcinoma cell lines MHCC-97H and HCC-LM3 were used. Cells were maintained in DMEM supplemented with 10% fetal bovine serum, 100 units/ml penicillin, and 100 µg/ml streptomycin, in a humidified atmosphere with 5% CO_2_ at 37°C.

### Reagents and treatments

Sorafenib (S1040, Selleck) was obtained from a reliable source and prepared as a stock solution. Recombinant human IL-22 (200-22, PeproTech) and antibodies for CD155 (ab267788, Abcam, UK), STAT3 (ab68153, Abcam, UK), p-STAT3(Y705) (ab267373, Abcam, UK), p-STAT3(S727) (ab219593, Abcam, UK), Ki-67 (GB13030-2, Servicebio, China), β-actin (ab6276, Abcam, UK) and GAPDH (ab8245, Abcam, UK) were obtained from reputable suppliers. Detailed information was available in **Supplementary Table 1**.

### Chromatin immunoprecipitation assay

As we previously reported, the ChIP assay was performed using the Pierce Magnetic ChIP Kit (Thermo Fisher Scientific) according to the manufacturer’s instructions (15). In brief, indicated cells were cross-linked using 1% paraformaldehyde and quenched by glycine solution. Then anti–STAT3 antibody and normal IgG (Millipore) were used for immunoprecipitation. ChIP-enriched DNA samples were quantified by real-time PCR to determine the specific binding sites (BS) of the *CD155* promoter region. The sequences of primers used for real-time PCR were summarized in **Supplementary Table 2**.

### Cell transfection

Initially, cells were seeded in culture plates and allowed to reach the appropriate confluency. We then performed the transfection using a suitable transfection reagent, adhering closely to the manufacturer’s protocol. The siRNA concentration and incubation time were meticulously optimized to ensure efficient gene knockdown while minimizing cytotoxic effects. After transfection, the cells were incubated for an optimal period to allow for effective gene silencing before further experimental assays were conducted. The siRNA sequences used for the transfection experiments were as follows: for STAT3: sense: 5’-GCACCUUCCUGCUAAGAUUTT-3’, anti-sense: 5’-AAUCUUAGCAGGAAGGUGCTT-3’; for CD155: sense: 5’-CUGUGAACCUCACCGUGUATT-3’, anti-sense: 5’-UACACGGUGAGGUUCACAGTT-3’; for the negative control (NC): sense: 5’-UUCUCCGAACGUGUCACGUTT-3’, anti-sense: 5’-ACGUGACACGUUCGGAGAATT-3’. Detailed information can be obtained from **Supplementary Table 3**.

### Immunohistochemistry assay

Formalin-fixed, paraffin-embedded HCC tissue samples were sectioned at a thickness of 4 µm. Sections were mounted on positively charged slides and dried overnight at 37°C. For deparaffinization, slides were immersed in xylene twice for 10 minutes each, followed by rehydration in a graded alcohol series (100%, 95%, 70%, and 50% ethanol) for 5 minutes each. For antigen retrieval, slides were submerged in 10 mM citrate buffer (pH 6.0) and heated in a microwave at high power for 15 minutes. After cooling to room temperature, the slides were rinsed in phosphate-buffered saline (PBS) for 5 minutes. Endogenous peroxidase activity was blocked by incubating the sections in 3% hydrogen peroxide for 10 minutes. Non-specific binding was minimized by incubating the slides in blocking solution (5% normal goat serum) for 1 hour at room temperature. The sections were then incubated overnight at 4°C with primary antibodies at appropriate dilutions as recommended by the manufacturer. After washing in PBS, the sections were incubated with biotinylated secondary antibodies for 1 hour at room temperature. The signal was amplified using a streptavidin-biotin complex (SABC) and visualized with diaminobenzidine (DAB) substrate. The reaction was monitored under a microscope and stopped by rinsing the slides in water once the desired stain intensity was achieved. Sections were counterstained with hematoxylin for 1-2 minutes, rinsed in running tap water, and then dehydrated in an ascending series of alcohols, cleared in xylene, and mounted with a xylene-based mounting medium. The stained slides were examined under a light microscope. Images were captured using a digital camera system attached to the microscope. The staining intensity and percentage of positive cells were evaluated and quantified using image analysis software. A semi-quantitative scoring system was used to assess the level of protein expression in the tissue sections.

### Cell counting kit-8 assay

After the treatment period, Cell Counting Kit-8 reagent (ab228554, Abcam, UK) was used to assess cell viability. A volume of 10 μL of CCK-8 solution was added to each well containing 100 μL of culture medium. The plates were then incubated at 37°C in a humidified atmosphere with 5% CO_2_ for 1-4 hours, allowing sufficient time for the colorimetric reaction to occur. The absorbance at 450 nm, which correlates with the number of viable cells, was measured using a microplate reader. The background absorbance from wells containing medium and CCK-8 solution without cells was subtracted from all experimental readings.

### Tunel assay

After treatment, cells were harvested and fixed in 4% paraformaldehyde at room temperature for 1 hour. Following fixation, cells were washed with PBS and permeabilized with 0.1% Triton X-100 in 0.1% sodium citrate for 2 minutes on ice. For the TUNEL reaction, cells were incubated with the TUNEL reaction mixture containing terminal deoxynucleotidyl transferase (TdT) and fluorescein-Br-dUTP, following the manufacturer’s instructions (ab66110, Abcam, UK). This step was performed in a dark, humidified chamber at 37°C for 1 hour. The TdT enzyme catalyzes the addition of fluorescein- Br-dUTP at the 3’-OH ends of fragmented DNA, a hallmark of apoptosis. After the TUNEL reaction, cells were rinsed three times with PBS to remove unincorporated fluorescein- Br-dUTP. For nuclear counterstaining, cells were incubated with DAPI (4’,6-diamidino-2-phenylindole) for 5 minutes at room temperature. Finally, cells were washed with PBS and mounted with an anti-fade mounting medium. The stained cells were examined under a fluorescence microscope. TUNEL-positive cells, indicative of apoptosis, emit red fluorescence due to the incorporation of fluorescein- Br-dUTP, whereas the nuclei of all cells were stained blue with DAPI (16).

### Edu assay

HCC cell lines were cultured and treated according to experimental conditions. Post-treatment, cells were incubated with Edu (5-ethynyl-2’-deoxyuridine) for a duration recommended by the manufacturer, typically 2 hours. After Edu incorporation, cells were fixed with 4% paraformaldehyde and permeabilized with a buffer containing Triton X-100. The incorporated Edu was detected using a Click-iT reaction (ab219801, Abcam, UK), which labels Edu with a fluorescent dye. Post labeling, cells were counterstained with DAPI for nuclear visualization and mounted for fluorescence microscopy. Edu-positive cells were identified by their fluorescence. Fluorescent Edu-positive cells were counted, and the proliferation rate was calculated as the percentage of Edu-positive cells relative to the total cell count (17).

### Western blotting assay

Cells were lysed using a RIPA buffer containing protease and phosphatase inhibitors. The lysates were centrifuged, and the supernatant was collected. Total protein concentration was determined using a BCA Protein Assay Kit. Equal amounts of protein were separated by SDS-PAGE on a polyacrylamide gel and then transferred onto a PVDF membrane using a wet transfer system. The membrane was blocked with 5% non-fat milk in TBST (Tris-buffered saline, 0.1% Tween 20) for 1 hour at room temperature to prevent non-specific binding. The membrane was then incubated with primary antibodies against target proteins overnight at 4°C. After washing, the membrane was incubated with HRP-conjugated secondary antibodies for 1 hour at room temperature. The protein bands were visualized using an enhanced chemiluminescence (ECL) detection system. The membrane was exposed to an X-ray film or a digital imaging system to capture the signal.

### RNA isolation and quantitative real-time PCR

Total RNA was extracted using an RNA isolation kit (9767, Takara, Japan) according to the manufacturer’s instructions. The quality and concentration of RNA were assessed using a spectrophotometer. Reverse transcription was performed to synthesize cDNA from the extracted RNA. A typical reaction mixture included RNA, reverse transcriptase, dNTPs, reverse transcription buffer, and primers. The reaction was carried out in a thermal cycler under conditions specified by the reverse transcriptase enzyme used. Quantitative real-time PCR was performed using a qRT-PCR kit (RR092A, Takara, Japan) on a real-time PCR system. The reaction mixture included cDNA, forward and reverse primers for the target genes (IL-22, STAT3, CD155), and a SYBR Green or TaqMan probe. PCR conditions were optimized for each primer pair, typically involving an initial denaturation step followed by cycles of denaturation, annealing, and extension.

### Subcellular protein fraction

Cells were grown to the desired confluence, treated as per experimental requirements, and then harvested by trypsinization. Cells were resuspended in a hypotonic lysis buffer and incubated on ice for 15 minutes to allow swelling. NP-40 was added to a final concentration of 0.5% to lyse the cell membrane, followed by gentle vortex. The lysate was centrifuged at high speed (14,000 g for 30 seconds) to separate the cytoplasmic fraction from the nuclear pellet. The nuclear pellet was washed once with the hypotonic buffer and then resuspended in nuclear extraction buffer (containing a higher salt concentration and protease inhibitors). This suspension was incubated on ice for 30 minutes with intermittent mixing, followed by centrifugation at high speed (14,000 g for 10 minutes) to obtain the nuclear extract. Protein concentrations in both cytoplasmic and nuclear fractions were determined using a suitable assay, such as the BCA Protein Assay, to ensure equal loading for subsequent analyses. To confirm the efficacy of the separation, Western blotting was performed using markers specific for the cytoplasm (GAPDH, ab8245, Abcam, UK) and nucleus (Histone H3, AF6359, Affinity, USA) (18).

### Luciferase reporter assays

The plasmids were constructed by VectorBuilder (Guangzhou, China). The renilla luciferase (Rluc) was placed in front and the firefly luciferase (Luc) was placed in the back. The sequence of STAT3 response elements was amplified and inserted in the middle of Rluc and Luc. The plasmids were transfected using Lipofectamine 3000 (Invitrogen, Carlsbad, CA, USA) in accordance with the manufacturer’s instructions. After culture for 48 hours, the luciferase activity was evaluated using the Duo-Luciferase HS Assay Kit (GeneCopoeia, CA, USA). The results were calculated by using Luc/Rluc activity (19).

### Lactate dehydrogenase release assay

NK cells are cultured and then co-incubated with target cells at various effector-to-target (E:T) ratios. The co-incubation is typically carried out for a specific duration, usually ranging from 4 to 6 hours, depending on the experimental design. Post co-incubation, the culture plates are centrifuged, and supernatants are collected carefully to avoid disturbing the cell pellet. LDH activity in the supernatant is measured using an LDH release assay kit (ab102526, Abcam, UK). This usually involves adding a substrate that reacts with LDH to produce a colorimetric or fluorometric signal. The reaction is incubated for a time recommended by the kit’s protocol, after which a stop solution is added if required. Maximum LDH release controls and spontaneous LDH release controls are included to calibrate the assay. Background LDH activity from NK cells alone is also measured to ensure specificity (20).

### HCC-NK cell co-culture system and calcein AM staining assay

As previously reported (21), calcein AM cytotoxicity assay was performed by using the calcein AM staining kit according to the manufacturer’s instructions (CA1630, Solarbio, China). Briefly, 1 × 10^5^ HCC cells were seeded into a 96-well plates which were pre-coated with poly-Lornithine. Then, the culture medium containing 1 μM calcein AM was added into the plates and incubated at 37 °C for 20 min. After that, NK-92MI cells were seeded into the plates at indicated ratio for 2 hours. Recombinant human IL-22 was added at a concentration of 10 ng/ml and sorafenib was added at 10 μM for specific experiments. Subsequently, the cells were washed using PBS and images were acquired using fluorescence microscopy.

### *In vivo* xenograft assay

For *in vivo* xenograft assay, 5-week old BALB/c nude mice (purchased from Charles River, Beijing, China) were used and randomly allocated to treatment or control groups (5 per group). The animals received humane care in accordance with the NIH Guide for Care and Use of Laboratory Animals and experiments with mice were performed under a well described protocol which has been approved by Institutional Animal Care and Use Committee (IACUC) of the Chongqing University. 2 million MHCC-97H cells were subcutaneously implanted into the axilla of mice. Tumor size was measured using the formula of length × width × width/2. After two weeks, the mice were fed with sorafenib (30 mg/kg body weight, three times per week), while IL-22 (2 mg/kg body weight, three times a week) was injected intraperitoneally. The mice were sacrificed at fourth week after implantation. The xenografts were harvested and subject to measurement of tumor weight, IHC staining (22).

### Microarray source and in silico immune cell infiltration analysis

The microarray dataset from the phase 3 STORM trial comparing sorafenib with placebo as adjuvant treatment of HCC was downloaded from the Gene Expression Omnibus (GEO). GSE109211 contains Illumina HumanHT-12 WG-DASL V4.0 expression data from formalin-fixed paraffin-embedded (FFPE) tissue blocks of 140 HCC patients. 67 samples treated with sorafenib were included in current analysis. Microenvironment Cell Populations-counter (MCP-counter), a transcriptomic markers-based method reliably portraying the cellular heterogeneity landscape of tissue expression profiles, was performed to explore immune cell Infiltration. The proportion of NK cells and cytotoxic lymphocyte score were obtained for each sample per instruction. Bioinformatics analysis were performed by R software (23).

### Statistical analysis

In compiling experimental data from cellular, molecular, and *in vivo* studies, accuracy in data entry and normalization is crucial. The data were presented as the mean ± standard deviation (SD). All experiments were performed at least three replicates unless otherwise noted. Unpaired two-tailed Student’s t-test was utilized for two-sample comparisons and a two-way ANOVA was used for comparing two groups. We also included Cox regression analysis for survival data and log-rank tests for comparing survival curves. Chi-square tests were used for assessing categorical data associations. Advanced statistical methods such as regression, multivariate analysis, or Kaplan-Meier survival analysis were applied for complex data. All analyses were performed using statistical software SPSS (version 23.0) and GraphPad Prism (version 8.0). A two-tailed P value less than 0.05 was considered as statistically significant.

## Results

### Overexpression of IL-22 correlates with poor prognosis in HCC

**Figure 1:**
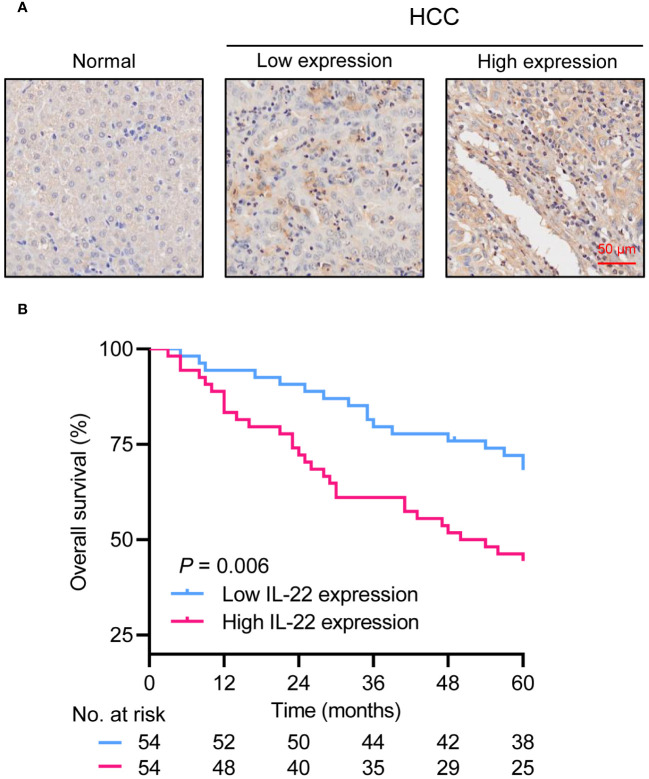
Overexpression of IL-22 in HCC correlates to poor prognosis in patients following liver resection. **(A)** Representative image of IHC staining in normal liver tissue (left) and HCC tumor tissue (right). Scale bar, 50 μm. **(B)** Kaplan-Meier survival curves demonstrating the relationship between IL-22 expression levels and overall survival in HCC patients. HCC, hepatocellular carcinoma. The log-rank test was used to determine the significance of difference between these two groups.

In our HCC resection cohort, analysis of HCC tissue samples revealed a significantly higher expression of IL-22 in tumor tissues compared to adjacent non-tumor tissues (**Figure 1A**). Combining the analysis of IL-22 expression levels with clinical data, we found that patients with high IL-22 expression presented with larger tumor diameters, more frequent microvascular invasion, and were more likely to be at an advanced stage of the disease (Stage III) at diagnosis (**Table 1**). Patients with high IL-22 expression were associated with significantly worse overall survival compared to those with low IL-22 expression, as determined by Kaplan-Meier analysis (**Figure 1B**). Univariate and multivariate Cox regression analyses suggested that elevated IL-22 expression was independent risk factor for overall survival after curative resection (**Table 2**).

**Table 1:** Association between IL-22 expression and patient characteristics.

Variables	No. of patients	IL-22 expression		P-value
		Low	High	
Age, year				
≤60	72 (66.7)	34 (63.0)	38 (70.4)	0.541
>60	36 (33.3)	20 (37.0)	16 (29.6)	
Gender				
Male	96 (88.9)	47 (87.0)	49 (90.7)	0.761
Female	12 (11.1)	7 (13.0)	5 (9.3)	
AFP, ng/ml				
≥400	69 (63.9)	37 (68.5)	32 (59.3)	0.423
<400	39 (36.1)	17 (31.5)	22 (40.7)	
Tumor size, cm				
≤5	62 (57.4)	38 (70.4)	24 (44.4)	0.007
>5	46 (42.6)	16 (29.6)	30 (55.6)	
Tumor number				
Single	86 (79.6)	49 (90.7)	37 (68.5)	0.008
Multiple	22 (20.4)	5 (9.3)	17 (31.5)	
Differentiation				
Well/Moderate	65 (60.2)	34 (63.0)	31 (57.4)	0.694
Poor	43 (39.8)	20 (37.0)	23 (42.6)	
Cirrhosis				
Absent	34 (31.5)	17 (31.5)	17 (31.5)	1.000
Present	74 (68.5)	37 (68.5)	37 (68.5)	
Vascular invasion				
Absent	105 (97.2)	53 (98.1)	52 (96.3)	1.000
Present	3 (2.8)	1 (1.9)	2 (3.7)	
Microvascular invasion				
Absent	67 (62.0)	40 (74.1)	27 (50.0)	0.017
Present	41 (38.0)	14 (25.9)	27 (50.0)	
TNM stage				
I- II	85 (78.7)	48 (88.9)	37 (68.5)	0.011
III	23 (21.3)	6 (11.1)	17 (31.5)	

TNM, tumor-node-metastasis; SD, standard deviation; CI, confidence interval.

**Table 2:** Univariate and multivariate Cox regression analysis of factors associated with overall survival in HCC patients.

Variables	Univariate			Multivariate		
	HR	95% CI	*P*	HR	95% CI	*P*
Age	0.625	0.324-1.204	0.145			
Gender (F/M)	0.669	0.240-1.866	0.443			
AFP (≥400/<400)	1.421	0.793-2.545	0.239			
Tumor size (>5/≤5)	1.075	1.002-1.153	0.044	1.029	0.953-1.111	0.469
Tumor number (multiple/single)	1.003	0.497-2.012	0.999			
Differentiation (Poor/Well-Moderate)	1.123	0.627-2.011	0.698			
Cirrhosis	1.526	0.792-2.941	0.192			
Macrovascular invasion	0.724	0.100-5.243	0.749			
Microvascular invasion	1.955	1.103-3.468	0.022	1.578	0.851-2.927	0.147
TNM stage (III/I-II)	1.371	0.712-2.642	0.359			
IL-22 expression (high/low)	2.209	1.217-4.011	0.009	1.888	1.008-3.537	0.047

TNM, tumor-node-metastasis; HR, hazard ratio; CI, confidence interval; F, female; M, male.

Then we explored the potential effect of IL-22/STAT3 signaling to discriminate sorafenib outcomes (non-responder or responder) in the phase 3 STORM trial comparing sorafenib with placebo as adjuvant treatment of HCC. Unfortunately, in the cohort of patients received sorafenib administration, IL-22 mRNA levels seem slightly lower in non-responder, rather than we would have assumed that IL-22 mRNA levels were expected to be higher in non-responder. Since we always keep in mind that a single gene mRNA level detected by microarray is not always consistent with its protein level, we tried to validate STAT3 signaling targets by multigene signatures. We classified validated STAT3 target genes into three categories: (1) genes associated with proliferation and survival: *BCL2L1*, *BCL2*, *BIRC5*, *CCND1*, *MYC*, *MCL1*, *TP53*, *CDKN1A*, *FAS*, *HSPA4*, *HSP90AA1*, *HSP90AB1*, *SLC22A1*, *POU5F1*, *JUNB*, *NANOG*, *SOX2*, *TNFRSF1A*, *ABCC1*, *BECN1*, *PIK3C3*, *BNIP3*, and *AKT1*; (2) genes associated with metastasis and angiogenesis: *MMPs* (1–3, 7, 9), *TWIST1*, *VIM*, *TIMP1*, *RHOA*, *ZEB1*, *ZEB2*, *SNAI1*, *HIF1A*, *CDH1*, *VEGFA*, *FGF2*, *HGF*, *FSCN1*, *RHOU*, *LCN2*, and *SAA1*; (3) genes associated with immunomodulation and inflammation: *IL6*, *IL10*, *CD274*, *TGFB1*, *ICAM1*, *IL11*, *IL17A*, *IL21*, *IL23A*, *PTGS2*, *CXCL12*, *IL12A*, *IFNB1*, *IFNG*, *CCL5*, *CXCL10*, *CD80*, *CD86*, *IL1B*, *CCL2*, *NOS2*, and *STAT1*. Hierarchical cluster analysis based on gene expression profiles well identified sorafenib outcomes (responder or non-responder) in each STAT3 target genes signature category (**Supplementary Figure 1**). Then we applied in silico immune cell infiltration analysis, results suggested that sorafenib non-responders had significantly lower NK cell infiltration and total cytotoxicity scores (**Supplementary Figure 2**). Collectively, the findings supported a hypothesis that STAT3 signaling was pivotal in promoting sorafenib resistance in scenario of adjuvant treatment of HCC.

### IL-22 enhances sorafenib resistance in HCC cells

**Figure 2:**
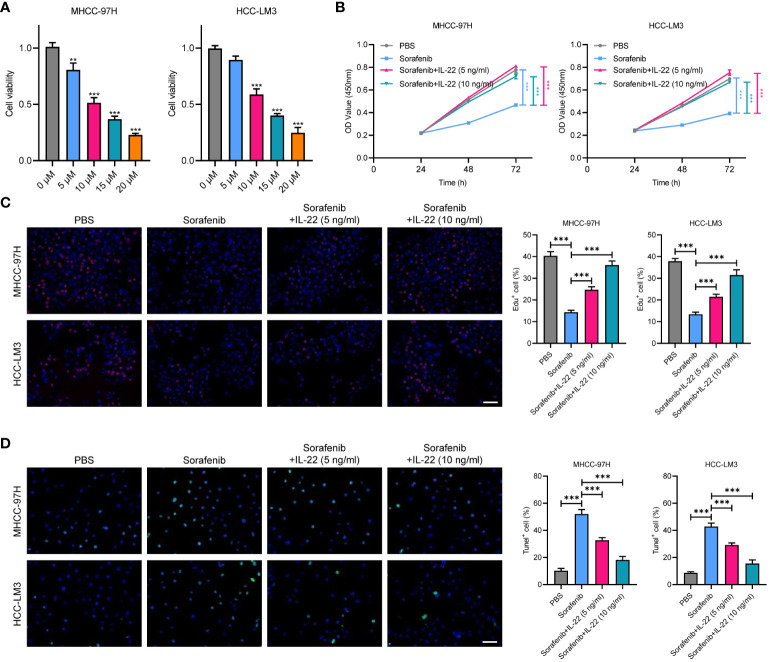
IL-22 promotes sorafenib resistance in HCC. **(A)** Analysis of cell viability in MHCC-97H and LM3 cells treated with various concentration of sorafenib (0 μM, 5 μM, 10μM, 15μM, and 20μM). **(B)** Growth rates of MHCC-97H and LM3 cells treated with PBS, Sorafenib (10μM), sorafenib + IL-22 (5 ng/ml), and sorafenib + IL-22 (10 ng/ml) using the CCK-8 assay. **(C)** Edu assays showing cell viability in MHCC-97H and LM3 cells treated with PBS, Sorafenib, sorafenib + IL-22 (5 ng/ml), and sorafenib + IL-22 (10 ng/ml). Scale bar, 50 μm. **(D)** Tunel assays showing apoptotic ratios of MHCC-97H and LM3 cells treated with different conditions. HCC, hepatocellular carcinoma. Scale bar, 50 μm. The data were pooled from at least three independent replicates and presented as the mean ± SD. Two-way ANOVA (in B) and unpaired two-tailed Student’s t test were used to determine the significance of differences between the indicated groups where applicable. **p < 0.01, ***p < 0.001.

We explored the impact of IL-22 on sorafenib resistance in HCC using two cell lines, HCC-LM3 and MHCC-97H. These cells were treated with varying concentrations of sorafenib (0 μM, 5 μM, 10μM, 15μM, and 20μM), revealing an IC50 value of 10μM (**Figure 2A**). We further conducted functional assays by dividing the cells into four groups: a control group, a sorafenib-treatment group, and two groups treated with sorafenib in combination with IL-22 at concentrations of 5 ng/ml and 10 ng/ml. The results of the CCK8 assay indicated that while sorafenib reduced cell proliferation, the addition of IL-22 countered this effect (**Figure 2B**). Similarly, the Edu assays showed that IL-22 improved sorafenib-reduced cell survival, which was also more effectively at a higher concentration (**Figure 2C**). Furthermore, the tunel assays suggested that IL-22 decreased the number of apoptotic cells induced by sorafenib, with the improvement being more pronounced at higher IL-22 concentrations (**Figure 2D**). These findings collectively demonstrated the role of IL-22 in enhancing sorafenib resistance in HCC cells.

### IL-22 stimulates STAT3 signaling in HCC cells

**Figure 3:**
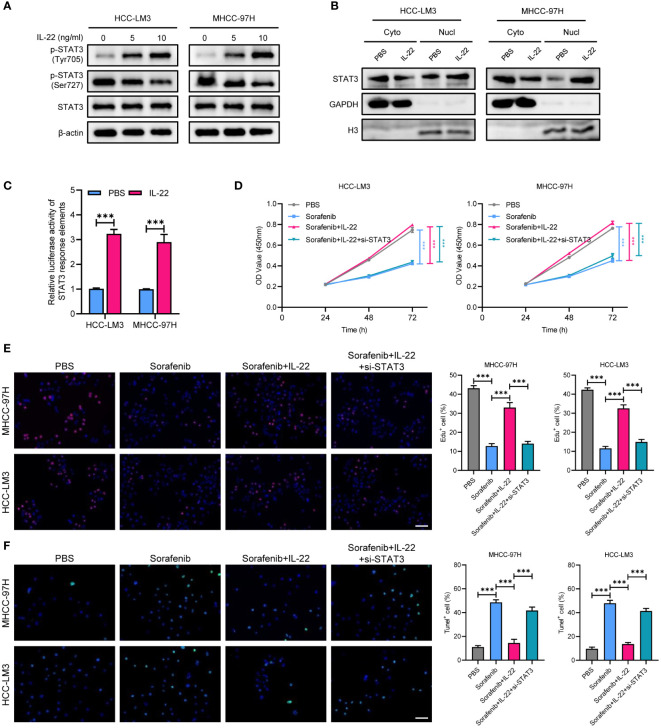
IL-22 enhances the activation of STAT3 signaling. **(A)** WB assays showing the expression of total STAT3, p-STAT3 (Tyr705), and p-STAT3 (Ser727) in MHCC-97H and LM3 cells treated with IL-22 at concentrations of 0 ng/ml, 5 ng/ml and 10 ng/ml, β-actin serves as control. **(B)** Subcellular fractionation assays showing the expression of STAT3 protein levels in the nucleus and cytoplasm of MHCC-97H and LM3 cells following IL-22 treatment, histone H3 and GAPDH serves as positive controls in the cytoplasm and nucleus, respectively. **(C)** Dual-luciferase reporter assays showing the activity of STAT3 response elements in MHCC-97H and LM3 cells treated with or without IL-22 (10 ng/ml). **(D)** CCK-8 assays measuring the cell growth rates of MHCC-97H and LM3 cells treated with PBS, sorafenib, sorafenib + IL-22, and sorafenib + IL-22 + STAT3 siRNA. **(E)** Edu assay evaluating the cell viability of MHCC-97H and LM3 cells. **(F)** Tunel assays assessing apoptosis rates in MHCC-97H and HCC-LM3 cells. Scale bar, 50 μm. The data were pooled from at least three independent replicates and presented as the mean ± SD. Two-way ANOVA (in D) and unpaired two-tailed Student’s t test were used to determine the significance of differences between the indicated groups where applicable. **p < 0.01, ***p < 0.001.

To explore the mechanism underlying IL-22-enhanced sorafenib resistance, we treated MHCC-97H and HCC-LM3 cell lines with IL-22 at concentrations of 5 ng/ml and 10 ng/ml. Then the expression of phosphorylated p-STAT3^Tyr705^ and p-STAT3^Ser727^, as well as total STAT3, were examined. The results indicated that IL-22 addition increased the expression of p-STAT3^Tyr705^ and decreased p-STAT3^Ser727^, with the magnitude of increase in p-STAT3^Tyr705^ being higher at increased IL-22 concentrations. However, the expression of total STAT3 remained constant across different concentrations of IL-22 (**Figure 3A**). Upon analyzing the nuclear and cytoplasmic fractions through western blotting, it was observed that the expression of p-STAT3 in the nuclear fraction was elevated after IL-22 treatment, confirming that IL-22 promoted phosphorylation of STAT3 and nuclear translocation (**Figure 3B**). The results of dual-luciferase reporter assays suggested an enhancement in STAT3 response elements activity following IL-22 treatment, confirming the IL-22-induced activation of STAT3 signaling (**Figure 3C**). CCK8 assays showed that cell proliferation was lowest in the sorafenib-treated group and highest in the (sorafenib + IL-22) group. Adding IL-22 to the sorafenib treatment increased cell proliferation, but this effect was reduced upon STAT3 knockdown (**Figure 3D**). Edu assays indicated that cell viability was lowest in the sorafenib-treated group and highest in the control group. The addition of IL-22 to sorafenib treatment enhanced cell viability, but this enhancement was diminished when STAT3 was knocked down (**Figure 3E**). Tunel assays revealed that the rate of apoptosis was highest in the sorafenib-treated group and lowest in the control group. The addition of IL-22 to sorafenib decreased the rate of apoptosis, but this protective effect against apoptosis was lessened with silence of STAT3 (**Figure 3F**). Collectively, the above results demonstrated that IL-22 enhanced sorafenib resistance through nuclear translocation of STAT3.

### STAT3 mediates IL-22-induced sorafenib resistance via upregulating the expression of CD155

**Figure 4:**
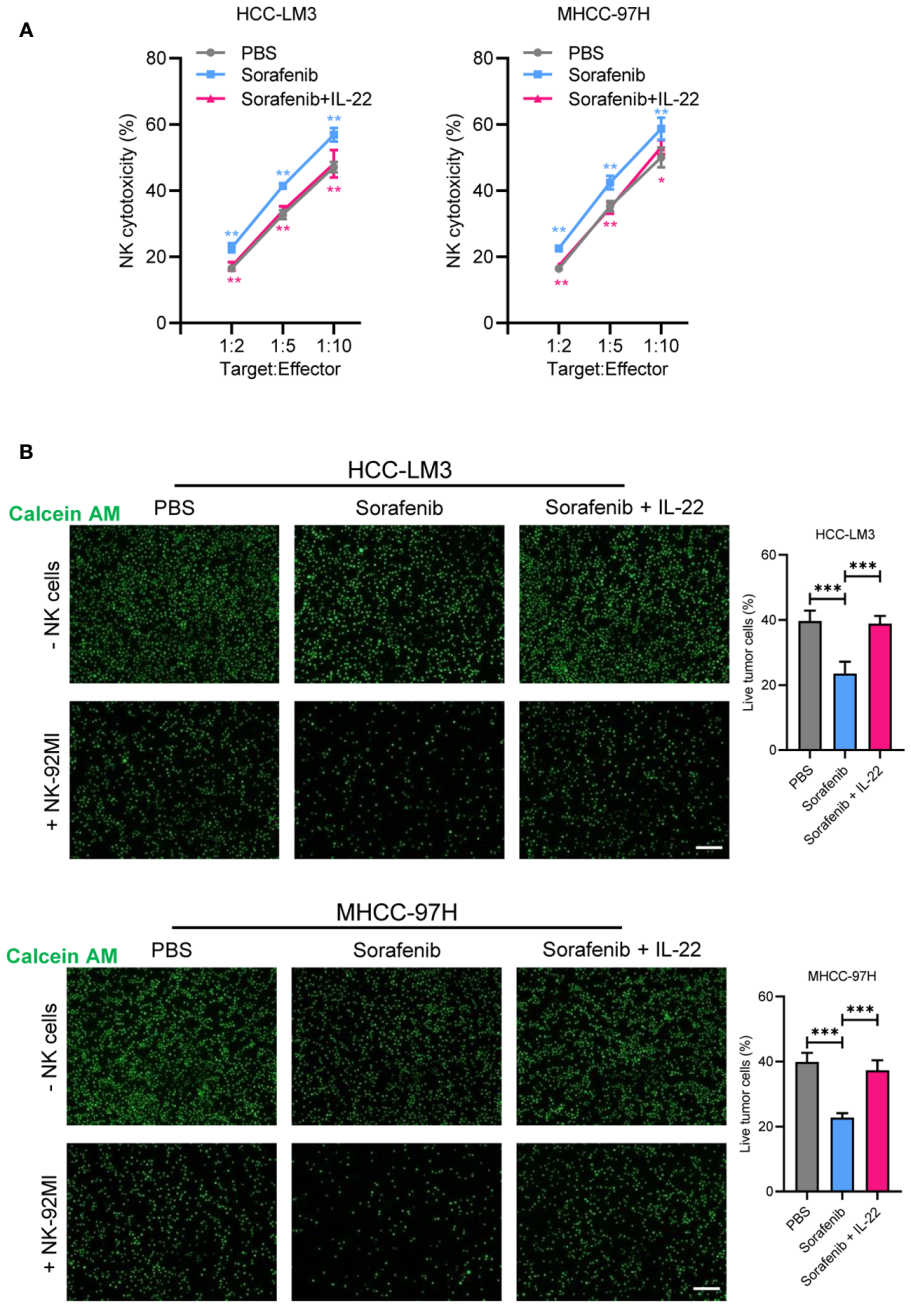
IL-22 regulated sorafenib resistance depended on NK-mediated cell killing. **(A)** HCC-LM3 and MHCC-97H cells were co-cultured with NK cells at different Target : Effector ratios for two hours. LDH activity was measured to calculate NK cell cytotoxicity. Cells were treated in different conditions. **(B)** HCC-LM3 and MHCC-97H cells were stained with calcein AM and seeded with NK cells at a Target : Effector ratio of 1:5. Representative images of live cells were acquired using fluorescence microscopy. Scale bar, 50 μm. The data were presented as the mean ± SD from at least three independent replicates. Unpaired two-tailed Student’s t test was used to determine the significance of differences between the indicated groups where applicable. *p < 0.05, **p < 0.01, ***p < 0.001.

**Figure 5:**
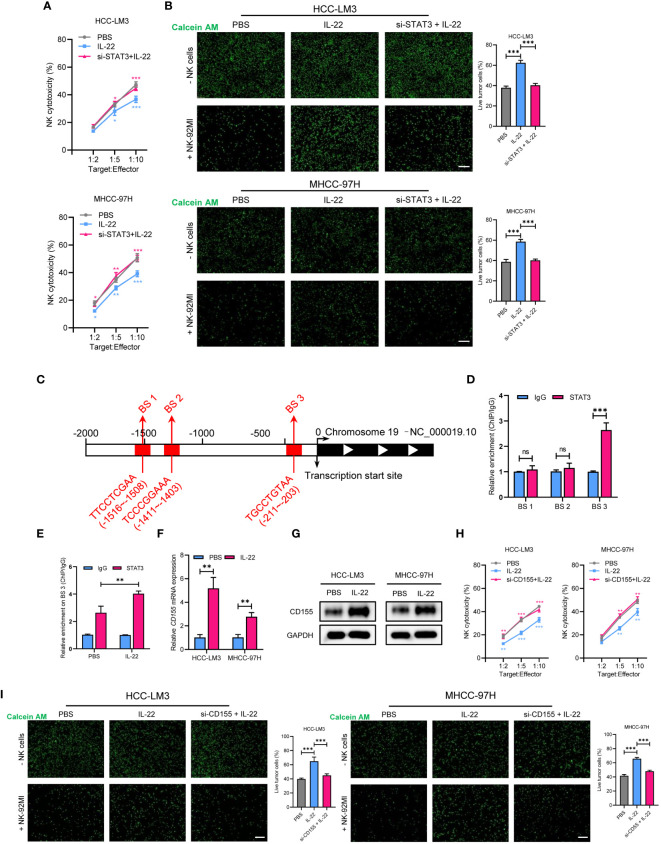
STAT3 regulates IL-22-increased sorafenib resistance in HCC via upregulation of CD155 expression. **(A)** MHCC-97H and HCC-LM3 cells were co-cultured with NK cells at different Target : Effector ratios for two hours. LDH activity was measured to calculate NK cell cytotoxicity. Cells were treated in different conditions. **(B)** MHCC-97H and HCC-LM3 cells were stained with calcein AM and seeded with NK cells at a Target : Effector ratio of 1:5. Representative images of live cells were acquired after co-cultured for two hours. Scale bar, 50 μm. **(C)** Schematic outlines of the predicted binding sites of STAT3 on the promoter region of CD155 gene. **(D)** ChIP assays of the enrichment of STAT3 on binding sites in the promoter region of *CD155*. **(E)** ChIP assays showing relative enrichment of STAT3 on BS3 in the promoter region of *CD155* gene in MHCC-97H cells. **(F)** qRT-PCR analysis showing the expression of CD155 mRNA expression in MHCC-97H and HCC-LM3 cells treated with or without IL-22 (10 ng/ml). **(G)** Western blotting assays showing the expression of CD155 protein in MHCC-97H and HCC-LM3 cells treated with or without IL-22 (10 ng/ml). **(H)** LDH activity was measured to calculate NK cell cytotoxicity. Cells were treated in the indicated conditions. **(I)** Calcein AM staining experiment analyzing MHCC-97H and HCC-LM3 cells treated in the indicated conditions. Scale bar, 50 μm. The data were presented as the mean ± SD from at least three independent replicates. Unpaired two-tailed Student’s t test was used to determine the significance of differences between the indicated groups where applicable. ns, not significant, *p < 0.05, **p < 0.01, ***p < 0.001.

As emerging evidence has reported the NK-regulated drug resistance, then we detected whether the effect of IL-22 on sorafenib resistance was depend on NK-mediated cell killing. Firstly, a co-cultured assay with sorafenib treatment was performed (**Figure 4**). The results of lactate dehydrogenase (LDH) release and calcium staining assays showed that live cell number was reduced upon co-cultured with NK-92 cells. After treating with sorafenib, a further decrease in the number of live tumor cells could be observed. While IL-22 treatment could reverse most of the effect and increase the number of live cells. Furthermore, the effect of IL-22 on NK-mediated cell killing could be subsequently abolished by treatment of STAT3 silence (**Figures 5A, B**). Previous studies have suggested that the expression of CD155, a regulator of NK-mediated cell killing, was regulated by STAT3 (24). To determine whether STAT3 regulated the activity of CD155 promoter and then influence NK-mediated cell viability, bioinformatics analyses were performed to predict the potential BS of STAT3 on *CD155* promoter region (JASPAR database, https://jaspar.elixir.no/). Three BS were found and verified by using ChIP assays (**Figure 5C**). Subsequent ChIP assays confirmed significantly high enrichment of STAT3 on BS3 in the promoter region of *CD155* gene (**Figure 5D**). Importantly, treatment of IL-22 significantly increased the enrichment of STAT3 on BS3, suggesting that IL-22 may indirectly affect the expression of CD155 at the transcriptional level (**Figure 5E**). Subsequently, we detected the expression of CD155 mRNA and protein levels after treating HCC cells with IL-22. The results shown that the expression of CD155 was upregulated upon IL-22 treatment (**Figures 5F, G**). Moreover, LDH release and calcium staining assays revealed that the activity of HCC cells was lowest upon addition of NK cells, significantly increased in the IL-22 treatment group, but decreased under the condition of IL-22 plus CD155 silence (**Figures 5H, I**). Taken together, these results suggested that IL-22 promoted sorafenib resistance via STAT3-regulated promoter activity of CD155, thus regulated NK-mediated cell killing.

### IL-22 promotes sorafenib resistance and tumor growth *in vivo*

**Figure 6:**
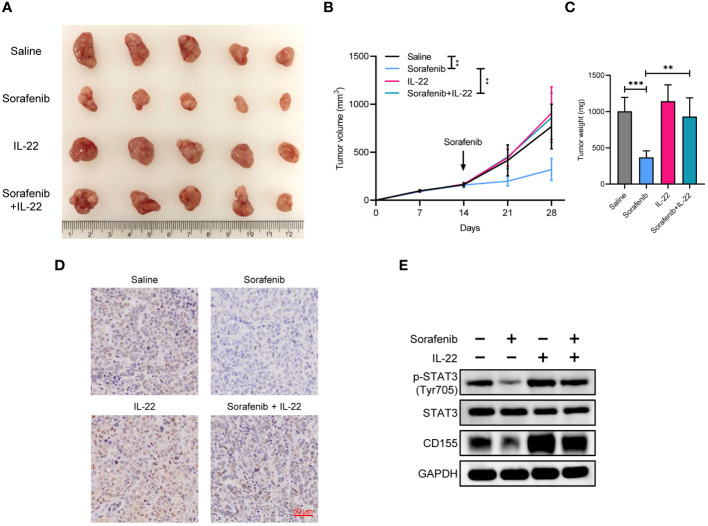
IL-22 promoted sorafenib resistance and tumor growth *in vivo*. **(A)** Gross tumor image showing the subcutaneous xenografts in nude mice injected MHCC-97H cells, which were subsequently treated with saline, sorafenib, IL-22, and sorafenib + IL-22. **(B)** Growth curves of subcutaneous xenografts. **(C)** Tumor weight of subcutaneous xenografts at 28 days post-injection were compared. **(D)** Representative images of immunohistochemistry staining of Ki-67 in xenografts. Scale bar, 50 μm. **(E)** Western blotting analyses evaluating CD155 expression levels in xenografts from each group. The data were presented as the mean ± SD of five biological replicates. Two-way ANOVA (in B) and unpaired two-tailed Student’s t test were used to determine the significance of differences between the indicated groups where applicable. **p < 0.01, ***p < 0.001.

To confirm whether IL-22 could promote sorafenib resistance *in vivo*, MHCC-97H cells were subcutaneously implanted into nude mice. After two weeks, the nude mice were fed with sorafenib and part of them were treated with IL-22 via intraperitoneal injection. Tumor volume was measured every week. Then the mice were sacrificed at the fourth week, the tumor weight were measured. The results showed that the group treated with sorafenib alone showed the slowest tumor growth and smallest tumor volume. The group treated with IL-22 alone showed no significant difference in volume and growth rate compared to the control group. Compared to the sorafenib group, the sorafenib + IL-22 group had faster tumor growth and larger tumor volume, indicating the effect of IL-22 on sorafenib resistance *in vivo* (**Figures 6A–C**). The IHC staining of Ki-67 confirming the cell proliferation efficacy of xenografts. Finally, the WB assays demonstrated that the expression of p-STAT3 (Tyr705) and CD155 decreased after treatment of sorafenib *in vivo*, which could be abolished by addition of IL-22. (**Figures 6D, E**).

## Discussion

HCC is a challenging and fatal kind of solid cancer. High heterogeneity, therapy resistance, post-resection recurrence, and metastasis are the major causes of dismal survival outcomes for HCC patients. During the past decade, sorafenib has been widely adopted as a first-line therapy for advanced HCC. Although approximately 30% patients with advanced HCC may benefit this therapy, it is unavoidable to develop drug resistance in cases (5). Therefore, there is still a pressing need to figure out the molecular and cellular mechanisms of HCC development and progression to develop more effective measures for treating HCC. In this study, we demonstrated that IL-22 was highly expressed in HCC and its elevated expression predicted worse patient survival prognosis. In addition, IL-22 significantly impaired sorafenib-mediated antitumor effect in HCC by activating tumor cell-intrinsic STAT3/CD155 axis to inhibit the cellular toxicity of sorafenib and NK cell -mediated killing to HCC cells. Thus, our findings indicated that IL-22 may serve as a potential therapeutic target for alleviating sorafenib resistance in HCC.

IL-22 is a member of the IL-10 cytokine family and plays a significant role in various biological functions. It can bind to heterodimer receptors comprising type 1 and type 2 receptor chains to activate multiple signaling pathways including STAT3, NF-κB, MAPK, and AKT. Although IL-22 is primarily produced by immune cells, its specific receptor IL-22R1 is expressed in nonimmune cells, such as hepatocytes and epithelial cells, highlighting its unique role in tissue protection, regeneration and pro-inflammatory effects​​​​ (25, 26). Remarkably, increasing evidence unveil that IL-22 participates in the development and progression of multiple types of cancers​​ (27). It has been reported that IL-22 could accelerate HCC progression through promoting tumor cell proliferation, migration and invasion, as well as inhibiting apoptosis (8). In this study, we found that IL-22 expression was highly expressed in HCC tissues versus adjacent liver tissues. Moreover, our data revealed a positive correlation of high IL-22 expression with larger HCC size, more frequent microvascular invasion, and advanced disease stage. Therefore, our findings and the literature evidence suggest that IL-22 plays an essential role in facilitating tumor progression and serve as a promising biomarker in HCC. However, its role in sorafenib resistance in HCC has never been extensively studied currently. Intriguingly, many studies focusing other types of tumors have shown that IL-22 is implicated drug resistance via complex mechanisms. For example, IL-22 confers lung cancer resistance to EGFR-Tyrosine Kinase Inhibitors through stimulating AKT and ERK signaling pathways​ (28)​. In colorectal cancer, IL-22 enhances autocrine expression of IL-8 in tumor cells via STAT3 activation, assisting tumor cells survive from FOLFOX chemotherapy (29). Analysis of transcriptomic data from a phase-3 STORM-trial indicated that STAT3 signaling activation may induce sorafenib resistant in HCC. Illuminated by these clues, here we further explored whether IL-22 involved in sorafenib resistance in HCC. As expected, our data from CCK8 and Edu assays showed that IL-22 addition significantly mitigated the direct inhibitory of sorafenib on HCC cell proliferation and survival. Furthermore, it decreased the number of apoptotic HCC cells induced by sorafenib. These results demonstrated that IL-22 contributed to the resistance of HCC cells to sorafenib. It was disclosed that IL-22 facilitated HCC growth and metastasis by activating STAT3 signaling pathway (8). Meanwhile, the aberrant tumor-intrinsic STAT3 activation has been found to augment the resistance of HCC cells to sorafenib (30–32). Based on these clues, we further tried to determine whether IL-22-mediated STAT3 activation involved in sorafenib resistance in HCC. As previously reported, STAT3 phosphorylation at Ser727 and Tyr705 differentially regulates the EMT–MET molecular subtypes of cells (33). The oncogenic effect of Tyr705 phosphorylation has been confirmed in various cancer types (34). However, the role of phosphorylation at Ser727 has not yet been well defined. Previous study has demonstrated that Ser727 phosphorylation on STAT3 was not necessarily a secondary event after Tyr705 phosphorylation and showed its role in the regulation of nuclear translocation of STAT3 in melanocytic cells (35). While Zhang et al. revealed oncogenic role of Tyr705 phosphorylation and suppressive effect of Ser727 phosphorylation on HCC progression (36). In this study, we found IL-22 treatment enhanced Tyr705 phosphorylation and decreased Ser727 phosphorylation of STAT3. The combined effect increased cytoplasmic STAT3 actively translocate into the nucleus, which also supported the interdependence of STAT3 tyrosine phosphorylation and serine phosphorylation in different cancer types (36). In addition, our data showed that IL-22 could prominently enhance proliferation and viability, and reduce apoptosis of sorafenib-treated HCC cells, and these effects were partly and markedly reversed by STAT3 knockdown. Collectively, these findings implied that IL-22 could confer HCC cells resistance to sorafenib-mediated cytotoxicity at least by activating STAT3 signaling pathway. Of note, a most recent study proved that IL-22 could upregulated anti-apoptotic and metastatic genes in HCC cells by triggering PI3K/AKT activation, resultantly promoting tumor cell proliferation and invasion (37). Furthermore, it has been shown that activation of PI3K/AKT signaling pathway also contributes to sorafenib resistance in HCC (38, 39). Therefore, it is possible that PI3K/AKT signaling pathway may also meditate IL-22-induced sorafenib resistance in HCC, which should be further studied in future.

NK cells, as innate lymphocytes, are crucial in immune surveillance against cancer, particularly in preventing metastases due to their ability to recognize and kill cancer cells (40–42). It has been revealed that sorafenib can enhance NK cell antitumor immunity in HCC, which closely correlates with its therapeutic efficacy (43). However, NK cell functions are often compromised by the tumor microenvironment of HCC (44). Interestingly, STAT3 activation in tumor cells can significantly impair NK cell antitumor immunity via multiple mechanisms (45). Therefore, we further investigated whether IL-22 regulated HCC cell sensitivity to NK cell cytotoxicity via STAT3 activation, which also took part in promoting sorafenib resistance. Our LDH release and calcium staining assays showed that IL-22 treatment prominently inhibited NK cell-mediated lysis of HCC cells, but STAT3 knockdown significantly mitigated this effect, indicating IL-22-induced STAT3 activation surely endowed HCC cell resistance to NK cell-mediated killing. It has been demonstrated that CD155, known as the poliovirus receptor (PVR), can interact with receptor CD226 on NK cells to impair NK cell function, thereby inducing tumor immune evasion (46). Coincidently, a recent study reported that IL-22 could upregulate CD155 expression in breast and lung cancer cells (13). Based on these clues, we hypothesized that IL-22 may reduce HCC cell sensitivity to NK cell-mediated killing via activating STAT3/CD155 axis. To test this hypothesis, we first explored whether and how STAT3 regulates CD155 expression in HCC cells. Our data showed that STAT3 could bind to the CD155 promoter and IL-22 treatment increased CD155 mRNA and protein expressions in HCC cells, and these effects were dramatically reversed by STAT3 knockdown. These results suggested that IL-22/STAT3 axis upregulated CD155 expression mainly at transcriptional level. Moreover, we observed that CD155 knockdown dramatically alleviated IL-22-induced resistance of HCC cells to NK cell cytotoxicity. Hence, our findings indicated that IL-22 could activate STAT3 to upregulate CD155 in HCC cells, resultantly repressing NK cell killing to HCC cells. Then, we employed HCC mice models to further determine IL-22-induced sorafenib resistance in HCC. It was observed that tumors grew much faster and had larger tumor volumes in mice simultaneously treated with sorafenib and IL-22 than those in mice exposed to sorafenib alone. Meanwhile, sorafenib significantly decreased the expression of p-STAT3 (Tyr705) and CD155 in HCC tissues, while IL-22 addition substantially attenuated these effects. These results further confirmed that IL-22 can promote sorafenib resistance in HCC via STAT3/CD155 axis.

## Conclusions

In conclusion, our study demonstrated that IL-22 was upregulated in HCC and its higher expression predicted worse patient survival prognosis. Moreover, IL-22 may contribute to sorafenib resistance of HCC through activating STAT3/CD155 signaling axis to decrease the sensitivities of tumor cells to sorafenib-mediated direct cytotoxicity and NK cell-mediated lysis. These findings deepen the understanding of how sorafenib resistance in HCC develops in terms of IL-22/STAT3 signaling pathway, and provide potential targets for overcome sorafenib resistance in patients with advanced HCC. However, our study is lack of clinical information of HCC patients with sorafenib treatment, which may discount the role of IL-22 in sorafenib resistance. Therefore, in future multi-center clinical studies should be performed to explore the prognostic significance of IL-22 expression in HCC patients treated with sorafenib and its correlation with the expressions of STAT3 and CD155 in the tumor tissues collected from those patients to validate our conclusion.

## Data availability statement

The original contributions presented in the study are included in the article/**Supplementary Material**. Further inquiries can be directed to the corresponding authors.

## Ethics statement

The studies involving humans were approved by Ethics Committee of Chongqing University Cancer Hospital. The studies were conducted in accordance with the local legislation and institutional requirements. The participants provided their written informed consent to participate in this study. The animal study was approved by Ethics Committee of Chongqing University Cancer Hospital. The study was conducted in accordance with the local legislation and institutional requirements.

## Author contributions

JC: Conceptualization, Data curation, Formal analysis, Methodology, Software, Writing – original draft, Writing – review & editing. SS: Data curation, Formal analysis, Funding acquisition, Methodology, Writing – original draft. HL: Data curation, Formal analysis, Methodology, Software, Writing – original draft. XC: Conceptualization, Formal analysis, Funding acquisition, Methodology, Writing – original draft. CW: Conceptualization, Supervision, Writing – original draft, Writing – review & editing.
