# Supplementary figures and images for: IL-22 signaling promotes sorafenib resistance in hepatocellular carcinoma via STAT3/CD155 signaling axis
Source: Front Immunol. 2024 Mar 25;15:1373321. doi: 10.3389/fimmu.2024.1373321 (PMC11003268; doi:10.3389/fimmu.2024.1373321)

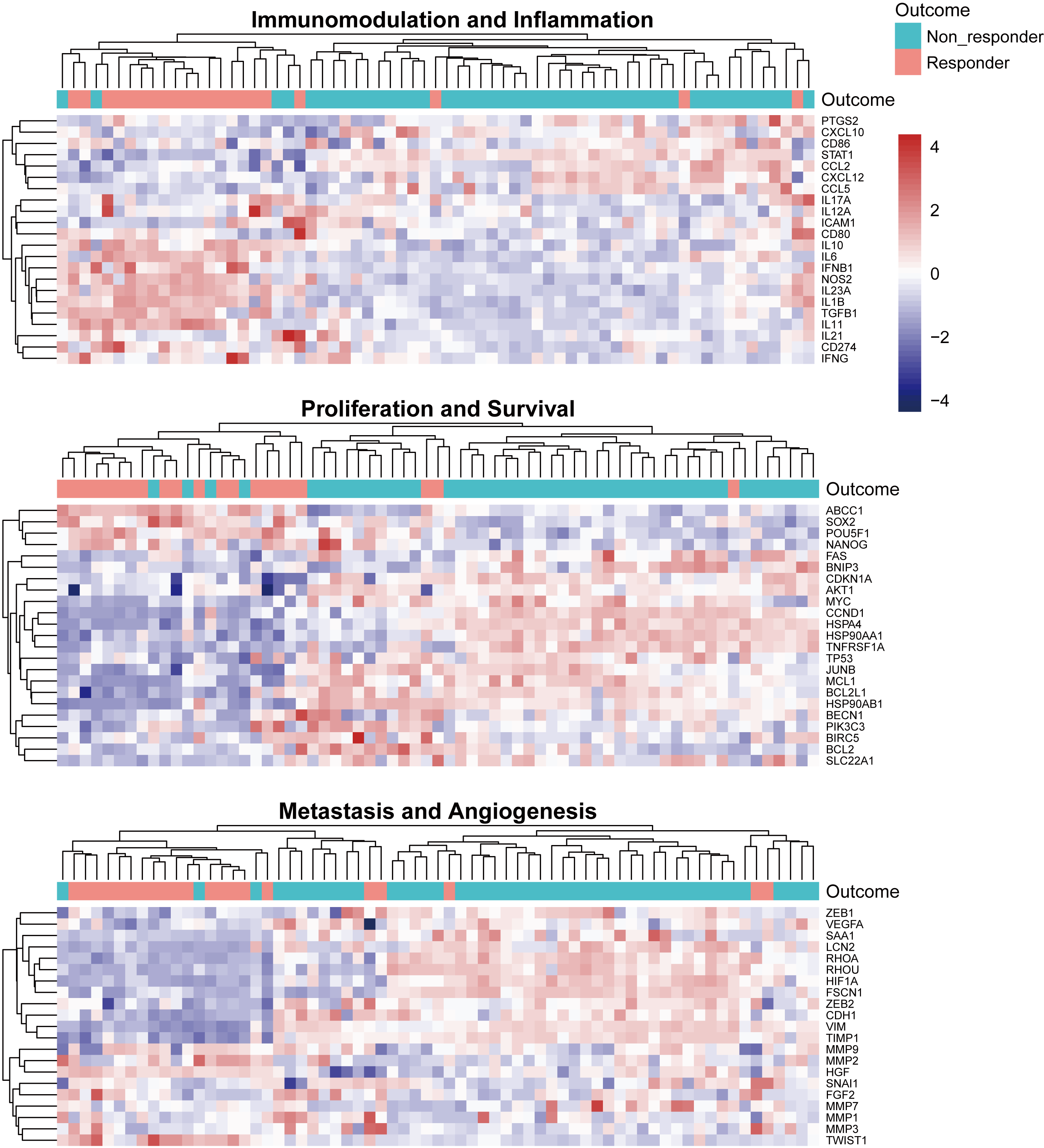

Supplement: Supplementary file 1 [file Image_1.tif]

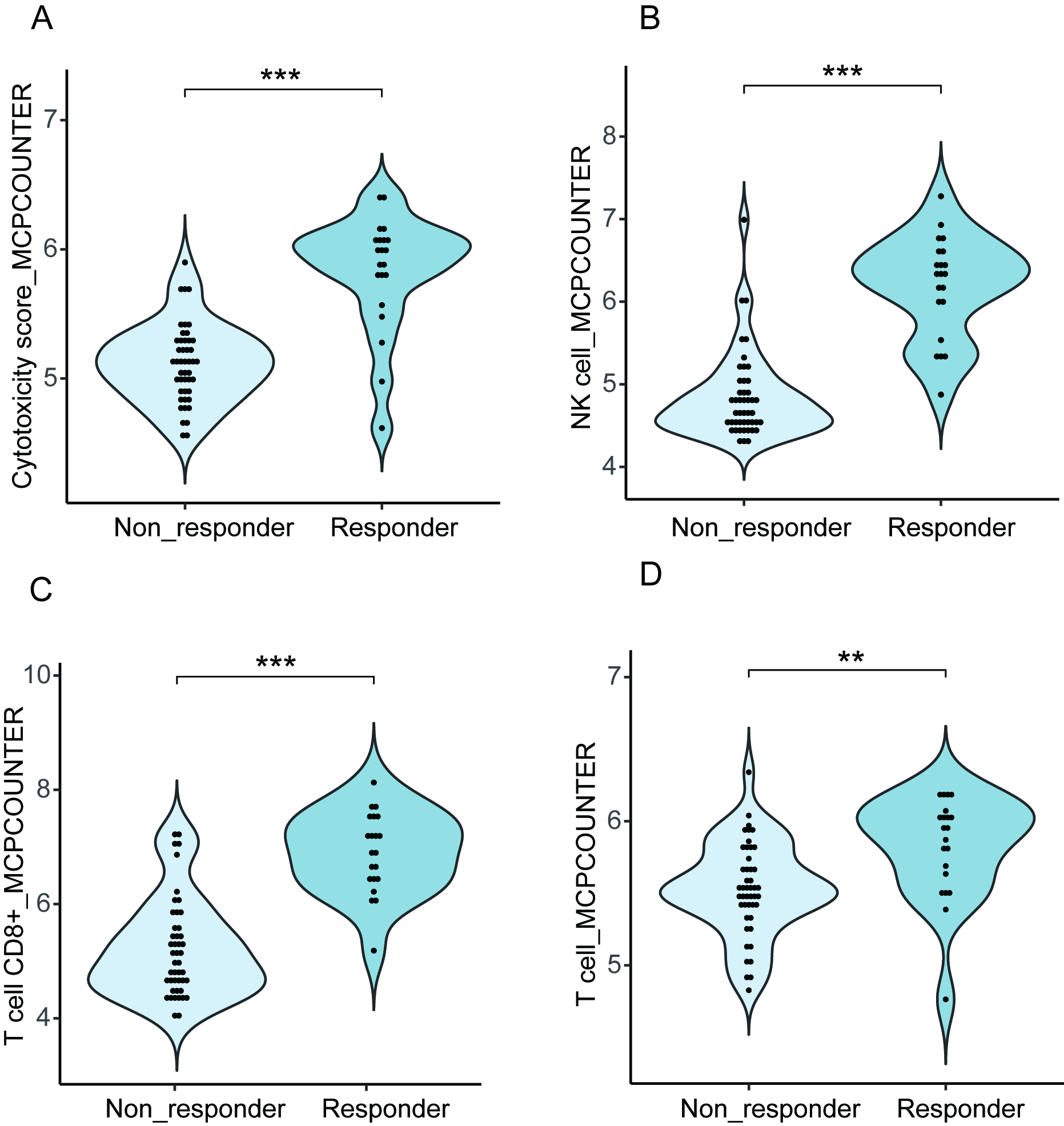

Supplement: Supplementary file 2 [file Image_2.tif]

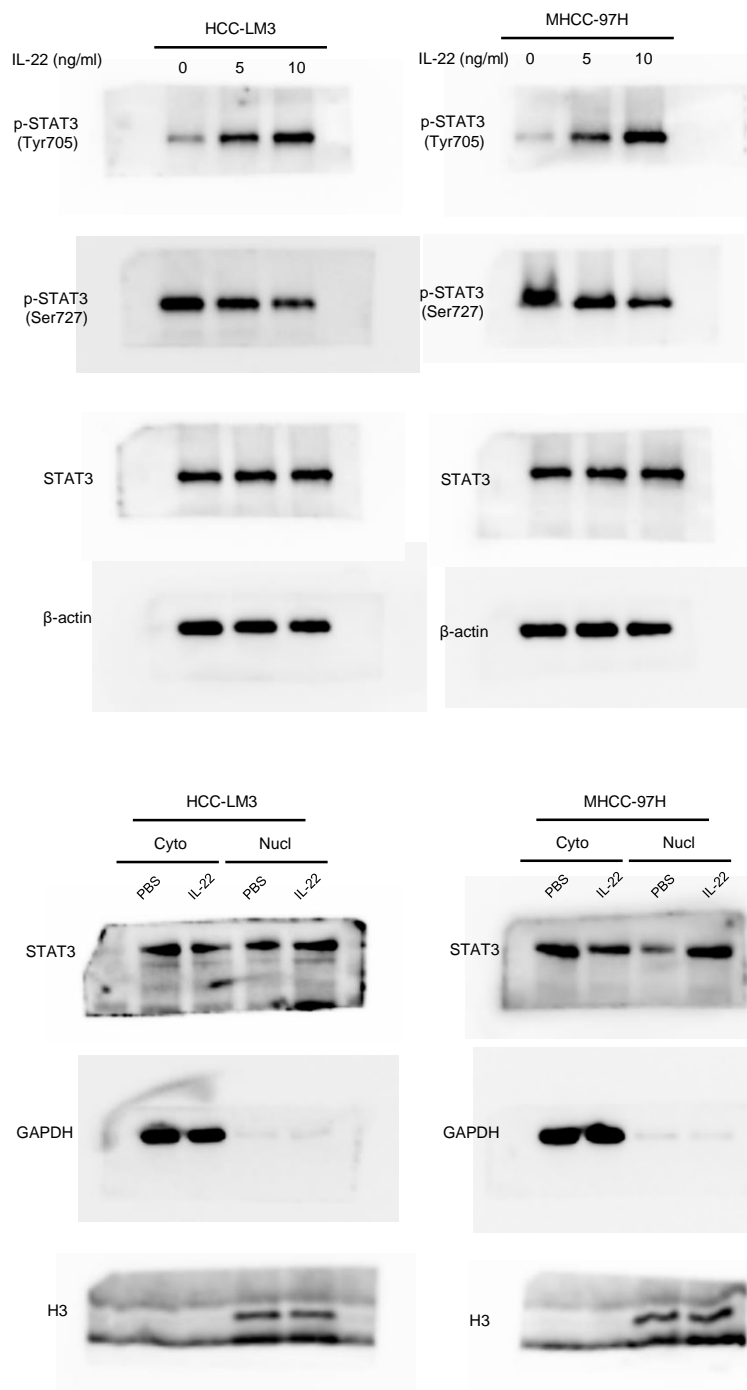

Refer to Figure 3

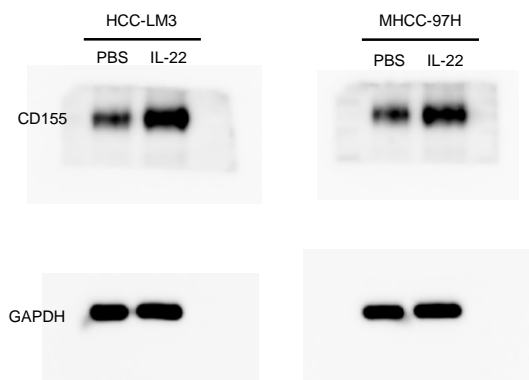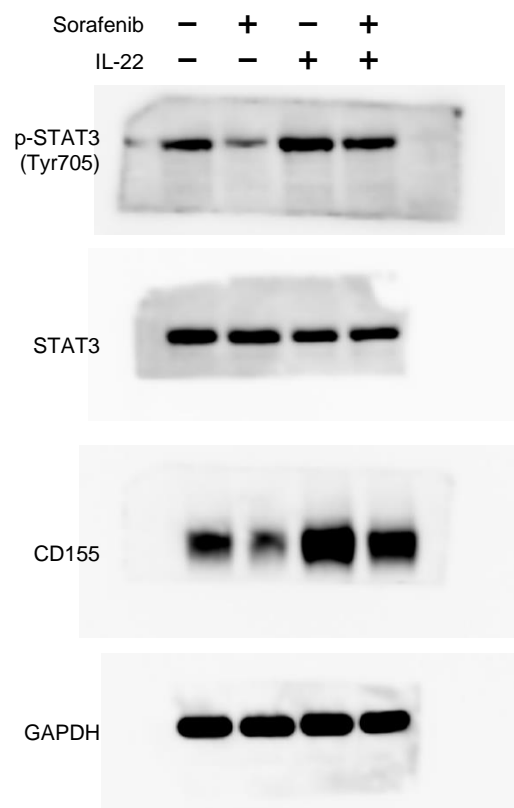

Refer to Figure 4 and Figure 5

Supplement: Supplementary file 3 [file Image_3.pdf]
