# Supplementary material for: IL-22 signaling promotes sorafenib resistance in hepatocellular carcinoma via STAT3/CD155 signaling axis
Source: Front Immunol. 2024 Mar 25;15:1373321. doi: 10.3389/fimmu.2024.1373321 (PMC11003268; doi:10.3389/fimmu.2024.1373321)
Supplement: Supplementary file 4 [file Table_1.docx]

Supplementary table 1. Primary antibodies used in this study.

| Antigens | Antibody sources | Species | Dilution | |
| --- | --- | --- | --- | --- |
| IL-22 | Abcam, ab134035 | Mouse monoclonal | 1:500 (IHC) |  |
| β-actin | Santa Cruz, sc-69879 | Mouse monoclonal | 1:1000 (WB) |  |
| Histone H3 | Affinity, AF6359 | Mouse monoclonal | 1:1000 (WB) |  |
| GAPDH | Abcam, ab8245 | Mouse monoclonal | 1:1000 (WB) |  |
| STAT3 | Abcam, ab68153 | Rabbit monoclonal | 1:1000 (WB) |  |
| p-STAT3 (y705) | Abcam, ab267373 | Rabbit monoclonal | 1:1000 (WB) |  |
| p-STAT3 (S727) | Abcam, ab219593 | Rabbit monoclonal | 1:1000 (WB) |  |
| CD155 | Abcam, ab267788 | Rabbit monoclonal | 1:1000 (WB) |  |
| Ki-67 | Servicebio, GB13030-2 | Rabbit monoclonal | 1:500 (IHC) |  |

Supplementary table 2. Primers used in this study.

| Primer names | Sequences |
| --- | --- |
| β-actin forward | GGGAAATCGTGCGTGACATTAAG |
| β-actin reverse | TGTGTTGGCGTACAGGTCTTTG |
| CD155 forward | GGCAACTACACCTGCCTGTT |
| CD155 reverse | AATCACCTGGCACTCAGACC |
| BS1 forward | GGGTTGGAAGGAACACGGT |
| BS1 reverse | CCCAACTCCATCCCCTTTCC |
| BS2 forward | GGAAAGGGGATGGAGTTGGG |
| BS2 reverse | TGGGTATGCTGAGCAGGAAC |
| BS3 forward | TGGTAGAGACAGGGTTTCGC |
| BS3 reverse | GCTCAAGTGATCCTCCCACC |

Supplementary table 3. Sequences of siRNA used in this study.

| Names | | Sequences |
| --- | --- | --- |
| STAT3 siRNA sense | 5’-GCACCUUCCUGCUAAGAUUTT-3’ | |
| STAT3 siRNA anti-sense | 5’-AAUCUUAGCAGGAAGGUGCTT-3’ | |
| CD155 siRNA sense | | 5’- CUGUGAACCUCACCGUGUATT-3’ |
| CD155 siRNA anti-sense | | 5’- UACACGGUGAGGUUCACAGTT-3’ |
| NC siRNA sense | | 5’-UUCUCCGAACGUGUCACGUTT-3’ |
| NC siRNA anti-sense | | 5’-ACGUGACACGUUCGGAGAATT-3’ |

**Supplementary figure legends**

**Supplementary figure 1**. Functional categorized STAT3 target genes signature associated with sorafenib response in sub-cohort of STORM trial with sorafenib treatment. Heatmap displaying the STAT3 signaling characteristics of sorafenib responders and non-responders.

**Supplementary figure 2**. Cytotoxic associated immune cell infiltration characteristics are significantly altered in the tumor tissue of sorafenib non-responders comparing to responders in sub-cohort of STORM trial with sorafenib treatment. The violin plots show the MCP-counter scores for cytotoxicity lymphocyte (A), NK cell (B), CD8+ T cell (C), T cell (D) of tumor tissue. **p < 0.01, ***p < 0.001.
